# Global prevalence of 4 neglected foodborne trematodes targeted for control by WHO: A scoping review to highlight the gaps

**DOI:** 10.1371/journal.pntd.0011073

**Published:** 2023-03-02

**Authors:** Rachel Tidman, Kaushi S. T. Kanankege, Mathieu Bangert, Bernadette Abela-Ridder

**Affiliations:** 1 Department of Control of Neglected Tropical Diseases, World Health Organization, Geneva, Switzerland; 2 World Organisation for Animal Health, Paris, France; 3 College of Veterinary Medicine, University of Minnesota, Saint Paul, Minnesota, United States of America; University of Zurich, SWITZERLAND

## Abstract

**Background:**

Foodborne trematodiases (FBTs) are a group of trematodes targeted for control as part of the World Health Organization (WHO) road map for neglected tropical diseases from 2021 to 2030. Disease mapping; surveillance; and capacity, awareness, and advocacy building are critical to reach the 2030 targets. This review aims to synthesise available data on FBT prevalence, risk factors, prevention, testing, and treatment.

**Methods:**

We searched the scientific literature and extracted prevalence data as well as qualitative data on the geographical and sociocultural risk factors associated with infection, preventive/protective factors, and methods and challenges of diagnostics and treatment. We also extracted WHO Global Health Observatory data representing the countries that reported FBTs during 2010 to 2019.

**Results:**

One hundred and fifteen studies reporting data on any of the 4 FBTs of focus (*Fasciola* spp., *Paragonimus* spp., *Clonorchis* sp., and *Opisthorchis* spp.) were included in the final selection. Opisthorchiasis was the most commonly reported and researched FBT, with recorded study prevalence ranging from 0.66% to 88.7% in Asia, and this was the highest FBT prevalence overall. The highest recorded study prevalence for clonorchiasis was 59.6%, reported in Asia. Fascioliasis was reported in all regions, with the highest prevalence of 24.77% reported in the Americas. The least data was available on paragonimiasis, with the highest reported study prevalence of 14.9% in Africa. WHO Global Health Observatory data indicated 93/224 (42%) countries reported at least 1 FBT and 26 countries are likely co-endemic to 2 or more FBTs. However, only 3 countries had conducted prevalence estimates for multiple FBTs in the published literature between 2010 to 2020. Despite differing epidemiology, there were overlapping risk factors for all FBTs in all geographical areas, including proximity to rural and agricultural environments; consumption of raw contaminated food; and limited water, hygiene, and sanitation. Mass drug administration and increased awareness and health education were commonly reported preventive factors for all FBTs. FBTs were primarily diagnosed using faecal parasitological testing. Triclabendazole was the most reported treatment for fascioliasis, while praziquantel was the primary treatment for paragonimiasis, clonorchiasis, and opisthorchiasis. Low sensitivity of diagnostic tests as well as reinfection due to continued high-risk food consumption habits were common factors.

**Conclusion:**

This review presents an up-to-date synthesis on the quantitative and qualitative evidence available for the 4 FBTs. The data show a large gap between what is being estimated and what is being reported. Although progress has been made with control programmes in several endemic areas, sustained effort is needed to improve surveillance data on FBTs and identify endemic and high-risk areas for environmental exposures, through a One Health approach, to achieve the 2030 goals of FBT prevention.

## Background

The cluster of selected foodborne trematodes (FBTs) listed by the World Health Organization (WHO) consists of 4 genera of trematodes (*Fasciola* spp., *Paragonimus* spp., *Clonorchis* sp., and *Opisthorchis* spp.) [1] Despite the prominent public health impacts of the FBTs, they remain listed as neglected tropical diseases (NTDs) by WHO. FBTs, like other NTDs, impact the most impoverished populations and lack the surveillance systems and tools to adequately ascertain their true burden [2,3]. FBTs are zoonotic diseases, with a complex lifecycle involving a primary intermediate snail host, and a secondary intermediate host for all except *Fasciola* spp. (crustaceans for *Paragonimus* spp., freshwater fish for *Clonorchis* sp. and *Opisthorchis* spp.), with humans becoming infected via the consumption of contaminated food [2].

FBT infections in humans result in a range of clinical symptoms including abdominal pain and fever, but could escalate to result in damage to internal organs including severe damage to the liver and lungs [1].

*Fasciola hepatica* is globally distributed, with *F*. *gigantica* distribution restricted to Africa and Asia. Infection sources are diverse and include contaminated food and water [4–6]. Fascioliasis most commonly results in inflammation of the bile ducts, gallbladder, and liver, resulting in liver fibrosis [5,6], but adult trematodes can also occur in the eyes and central nervous system, resulting in severe neurological and ocular symptoms [7]. Infection with *Paragonimus* spp. is acquired through the consumption of undercooked crab or crayfish and is found in Africa, Asia, and Latin America [4,5]. Adult flukes lodge in the lung tissue of the final host and can result in a chronic cough with bloody sputum, chest pain, and dyspnoea [5,6]. Ectopic infection in the brain is uncommon and may result in headaches, convulsions, and cerebral haemorrhages [5,6]. *Clonorchis sinensis* and *Opisthorchis viverrini* are largely confined to Asia, with infection acquired via the consumption of undercooked fish [5,6]. These parasites are classified as carcinogenic, as adult flukes can lodge in the bile ducts of the liver, causing inflammation of tissues and resulting in cholangiocarcinoma, a fatal bile duct cancer [5,6]. Infection with *Opisthorchis felineus* may result in acute abdominal pain due to gallbladder obstruction, and there is evidence that this is also carcinogenic [8].

FBTs have been reported in more than 70 countries worldwide [1]. The WHO Foodborne Disease Burden Epidemiology Reference Group attributes 200,000 illnesses and more than 7,000 deaths to FBTs annually [9]. In 2015, this was estimated to result in 1,066 thousand years disability-adjusted life years (DALYs) lost globally [10].

FBTs largely affect populations with poor sanitation and poor public health awareness [11,12]. The diversity of FBTs, range of clinical presentation, lack of sensitive diagnostic tools and dedicated surveillance systems, differences in host susceptibility, and different cultural food habits contribute to the underestimation and underreporting of these pathogens [2,6]. The true burden remains unknown as accurate prevalence data are scarce, and many endemic countries do not have national surveillance programmes in place [2].

FBTs are targeted for control as part of the WHO NTD road map 2021–2030, with mapping and surveillance and advocacy, capacity, and awareness building identified as critical actions required to reach the 2030 targets. A core component of this road map is the promotion of integrated One Health approaches in the development and implementation of NTD prevention and control programmes. Such One Health initiatives have already been implemented in several endemic areas, and while these initiatives have highlighted the complexity of FBT epidemiology and control, promising outcomes have been demonstrated which can be used as an example in other areas.

While several publications have introduced estimates of FBTs in 2012, up-to-date global estimates of foodborne trematodes focussing on prevalence data are needed to support the 2030 WHO NTD road map [6].

Considering these FBTs are confined to areas where the intermediate host species inhabit and the specific cultural or food habits of people leading to exposure to the pathogens, identifying the FBTs in relation to the geographical area is important. Furthermore, knowledge on co-endemicity of multiple FBTs in certain countries may inform planning campaigns of preventive chemotherapy against more than 1 FBT simultaneously, which could improve the cost-effectiveness of these campaigns [2,11,13–15]. Therefore, the overarching objective of this review is to bring together available data on reporting, prevalence, risk factors, prevention, testing, and treatment of the FBTs while identifying the potential for co-endemicity and data gaps at the country level.

## Methods

### Search strategy and selection criteria of published scientific literature

An initial broad-based search of PubMed, IRIS, Web of Science, Science Direct, and Cochrane electronic databases was performed using a combination of search terms, including each FBT and the terms “burden,” “prevalence,” “incidence,” and “cases.” A complete list of the specific search terms used can be found in Box A in S1 Supplementary material. No language restrictions were set, although all search terms were in English. All references published from January 2010 through February 2020 were included in the review. Additional records were identified through a snowballing approach, whereby the bibliographies of full text articles included from the initial literature search were screened, and any reference published after 2010 was reviewed using the same inclusion/exclusion criteria.

Records were initially screened based on title and abstract and were excluded if the abstract focused on an animal population and did not identify a human population of interest, and if the abstract did not mention 1 of the 4 trematode species of interest (*Fasciola* spp., *Paragonimus* spp., *Clonorchis* sp., and *Opisthorchis* spp.). Records were retained for full-text review if they identified a human population of interest and identified 1 or more of the 4 FBTs of interest. Full-text articles were evaluated for inclusion, and a second reviewer was consulted where there was ambiguity. Records were excluded if there was no prevalence or incidence recorded for a human population, for 1 or more of the 4 FBTs of interest. Records were also excluded if they did not specify the geographical area where data was collected, or if no diagnostic method was identified for determining prevalence. While the focus was on prevalence, 2 publications reported an incidence rate, and these records were included to highlight the presence of *O*. *felineus* captured in the literature. Extracted data included: parasite species and subspecies, geographical location, reported prevalence, time frame of study, diagnostic methods used, diagnostic challenges, treatments used, treatment challenges, environmental risk factors, sociocultural risk factors, and preventive factors.

### Extracting quantitative and presence only data

The prevalence of the 4 FBTs (i.e., fascioliasis, paragonimasis, clonorchiasis, and opisthorchiasis) were extracted.

Prevalence data at national level were scarce; therefore, prevalence studies of smaller spatial areas were recorded for the purpose of this review. Where multiple records were available for a specific country and parasite, the minimum and maximum prevalence were recorded. The full list of countries with WHO data and the selected studies and prevalence reported are included in Table A in S1 Supplementary material.

Presence only data on FBTs reported between 2010 and 2019 were extracted from the WHO Global Heath Observatory [16].

### Extracting qualitative data

We extracted qualitative data related to the risk and preventive factors associated with FBTs and grouped them into the following categories: environmental and sociocultural risk factors for infection, preventive measures, diagnostic methods and challenges, and treatment methods and challenges from each record. Qualitative data that were extracted included all risk and preventive factors that were discussed in each record and not only those which were the focus of the study.

### Mapping endemicity and co-endemicity of FBTs

Reported “Presence only” data from the WHO Global Health Observatory were mapped using Adobe Illustrator CS5, version 15.1.0 on WHO official template of world map, to represent the combinations of FBTs reported to the WHO Global Health Observatory. Countries that had reported more than 2 FBTs were considered to have potential for geographical co-endemicity. WHO records were compared with the records extracted through the review process to identify countries that has conducted epidemiological studies on the FBTs bteween 2010 and 2020 even if the records were not submitted to WHO.

## Results

### Literature review

**Fig 1:**
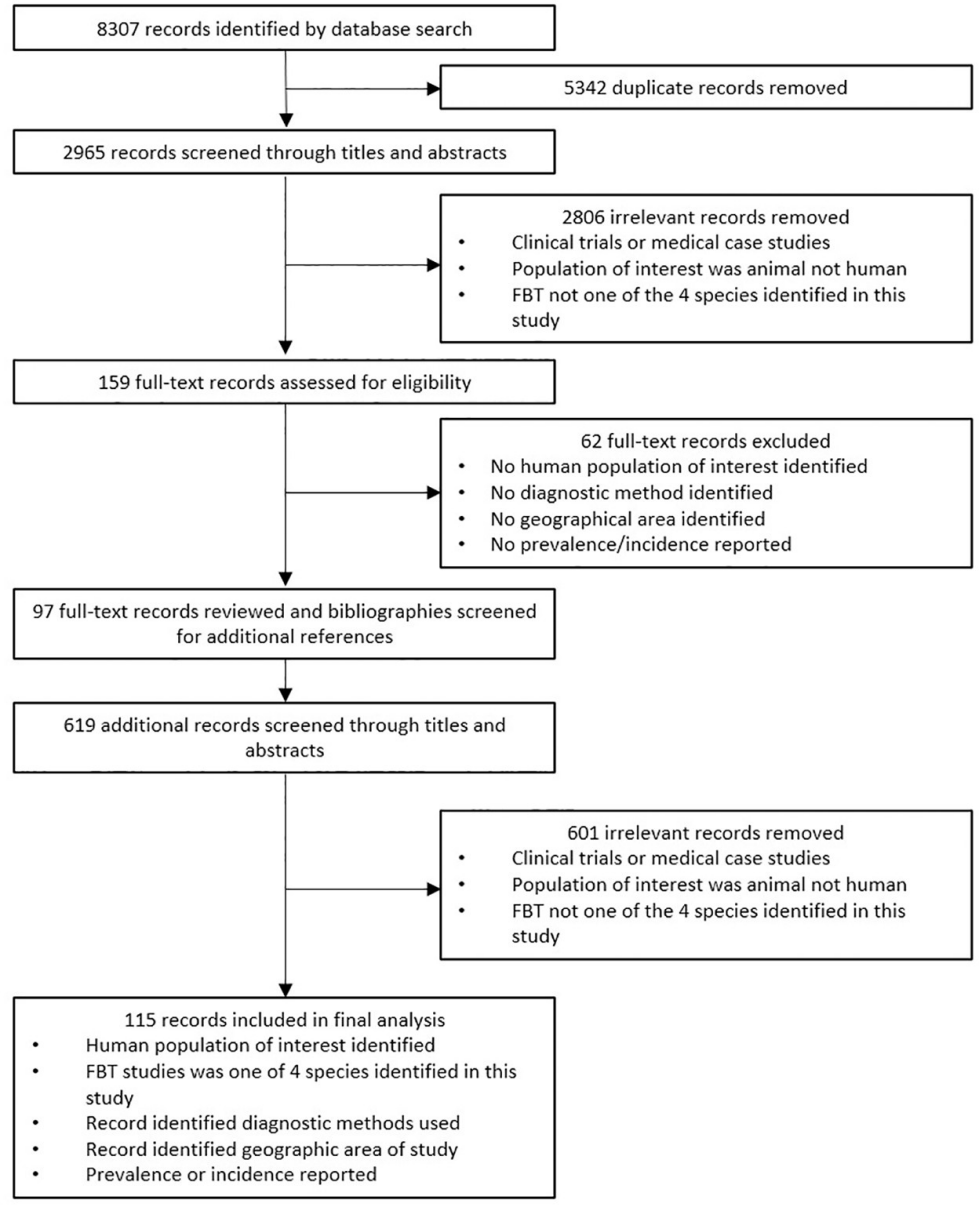
Flow diagram summarising the selection process of the studies in the review process.

After screening 8,926 records, 115 eligible full-text records providing prevalence data from 25 countries were included in the final dataset (Fig 1).

### Reported species, geography, and prevalence

Opisthorchiasis was the most recorded FBT globally, with 41 of the final full-text records reporting prevalence from 7 countries (Table 1). The highest recorded study prevalence for opisthorchiasis was in Lao People’s Democratic Republic, with a prevalence of up to 88.7% recorded in some areas. This study prevalence was overall the highest recorded of all the FBTs in this review.

**Table 1:** Study prevalence range of FBTs recorded in the literature from 2010–2020, presented by country and parasite.

Geographical region (and WHO region)	Country	Parasite	Study prevalence (range provided when more than 1 record)	Time frame of study	Study prevalence identified in most recent record	Range of sample sizes	No. of records included	Diagnostic methods used	References
Africa (AFR)	Ethiopia	*Fasciola* spp.	3.30%	2007–2008	3.3%	520	1	Parasitological (faecal)	[17]
	United Republic of Tanzania	*Fasciola* spp.	21.00%	2012–2013	21.0%	1,460	1	Parasitological (faecal)	[18]
	Cameroon	*Paragonimus africanus*	14.90%	2004–2006	14.9%	168	1	Immunological (serological) Parasitological (faecal and sputum)	[19]
Americas (AMR)	Argentina	*Fasciola hepatica*	11.90%	Not stated	11.9%	42	1	Immunological (serological) Parasitological (faecal)	[20]
	Brazil	*Fasciola hepatica*	1.80%	2013	1.8%	434	1	Immunological (serological) Parasitological (faecal)	[21]
	Bolivia	*Fasciola hepatica*	21.7–24.77%	2008	24.77%	436–437	2	Immunological (ELISA for coproantigen detection) Parasitological (faecal)	[22,23]
	Cuba	*Fasciola hepatica*	0.33%	2011–2012	0.33%	300	1	Parasitological (faecal)	[24]
	Haiti	*Fasciola hepatica*	6.50%	Not stated	6.5%	216	1	Immunological (serological)	[25]
	Mexico	*Fasciola hepatica*	5.78%	Not stated	5.78%	865	1	Parasitological (faecal)	[26]
	Peru	*Fasciola hepatica*	2.3–24.4%	2007–2017	10.10%	223–2,515	7	Immunological (serological) Parasitological (faecal)	[22, 27–32]
European (EUR)	Kyrgyzstan	*Fasciola hepatica*	1.90%	2009	1.9%	1,262	1	Parasitological (faecal)	[33]
	Turkey	*Fasciola hepatica*	0.04–5.6%	1977–2010	5.60%	1,600–69,633	2	Immunological (serological) Parasitological (faecal)	[34,35]
	Kazakhstan	*Opisthorchis felineus*	*incidence reported 7.4/100,000	1997–2011			1	Parasitological (faecal)	[36]
	Russian Federation	*Opisthorchis felineus*	*incidence reported 24.7/100,000	2011–2013			1	Parasitological (faecal)	[37]
Eastern Mediterranean (EMR)	Iran (Islamic Republic of)	*Fasciola* spp.	0.13–24.8%	2003–2016	2.60%	206–1984	12	Immunological (serological) Parasitological (faecal)	[38–49]
	Pakistan	*Fasciola* spp.	0.74–1.18%	2003–2005	0.74%	540–7,200	2	Parasitological (faecal)	[50,51]
Southeast Asia (SEAR)	India	*Paragonimus* spp.	6.16–11.0%	2008–2011	11.00%	624–4,371	2	Immunological (serological) Parasitological (faecal and sputum)	[52,53]
	Myanmar	*Opisthorchis viverrini*	0.7–9.3%	2015–2016	9.30%	364–2,057	2	Parasitological (faecal)	[54,55]
	Thailand	*Opisthorchis viverrini*	2.48–45.69%	1990–2017	17.00%	245–18,900	19	Immunological (urine ELISA) Parasitological (faecal)	[56–74]
Western Pacific (WPR)	Cambodia	*Opisthorchis viverrini*	4.6–47.5%	2006–2012	7.66%	228–32,201	6	Parasitological (faecal)	[75–80]
	China	*Fasciola gigantica*	8.8%	2011	8.8%	3,177	1	Immunological (serological) Parasitological (faecal)	[81]
		*Paragonimus* spp.	0.02–7.46%	2006–2013	0.02%	724–8,396	2	Immunological (serological) Parasitological (faecal)	[82,83]
		*Clonorchis sinensis*	0.03–59.6%	1989–2018	38.72%	718–356,629	13	Immunological (serological) Parasitological (faecal)	[82,84–95]
	Japan	*Paragonimus* spp.	8.5%	2001–2012	8.5%	5,200	1	Immunological (serological)	[96]
	Philippines	*Paragonimus westermani*	6.70%	2011–2013	6.7%	836	1	Parasitological (sputum)	[97]
	Lao People’s Democratic Republic	*Opisthorchis viverrini*	0.66–88.7%	2006–2015	87.90%	207–6,178	11	Parasitological (faecal)	[98–108]
	Republic of Korea	*Paragonimus westermani*	1.6–2.8%	1993–2011	1.60%	720–74,448	3	Immunological (serological)	[109–111]
		*Clonorchis sinensis*	0.2–28.2%	1993–2017	0.20%	231–99,451	15	Immunological (serological) Parasitological (faecal)	[109–123]
	Viet Nam	*Fasciola* spp.	5.90–7.8%	2012–2013	7.8%	1,612–10,084	2	Immunological (serological)	[124,125]
		*Paragonimus westermani*	5.40%	Not stated	5.4%	590	1	Immunological (serological) Parasitological (sputum)	[126]
		*Clonorchis sinensis*	16.47–22.72%	2009–2017	19.50%	400–1,857	4	Parasitological (faecal)	[127–130]
		*Opisthorchis viverrini*	11.40%	2015	11.4%	254	1	Parasitological (faecal)	[131]

*Incidence rates were reported, but no prevalence rate. These records were included to highlight that *O*. *felineus* presence was captured in the literature, in the specified time frame.

Fascioliasis had 36 records included in the final review, with the widest geographical range, representing 16 countries and all regions. The highest fascioliasis prevalence reported was 24.77% in Bolivia.

Clonorchiasis had 32 records included in the final review, reporting prevalence from 3 Asian countries. The highest recorded study prevalence for clonorchiasis was in China, with a prevalence of up to 59.6% recorded in some areas.

The least number of records were available on paragonimiasis, with only 11 papers reporting study prevalence from 7 countries, and the highest study prevalence of 14.9% was reported in Cameroon.

The following countries had the most available records on FBTs: 15 records for clonorchiasis in the Republic of Korea and 13 records in China, 12 records for fascioliasis in the Islamic Republic of Iran and 7 records in Peru, 19 records for opisthorchiasis in Thailand and 11 records in Lao People’s Democratic Republic.

### Global health observatory

Among the 224 countries and territories reporting data to the WHO Global Health Observatory from 2010 to 2019, 93/224 (42%) countries reported at least 1 of the 4 FBTs (i.e., 131/224 (58%) did not report any), and 2019 was the last available year of data as the process of reporting and validation of NTD surveillance data to WHO entails approximately 1-year lag in data availability. Fascioliasis was reported in 75/224 (33%), paragonimiasis in 44/224 (20%), clonorchiasis in 10/224 (4%), and opisthorchiasis in 7/224 (3%) countries. Among the reporting countries, 26 were co-endemic to 2 or more FBTs.

### Co-endemicity (2 or more FBTs identified)

**Fig 2:**
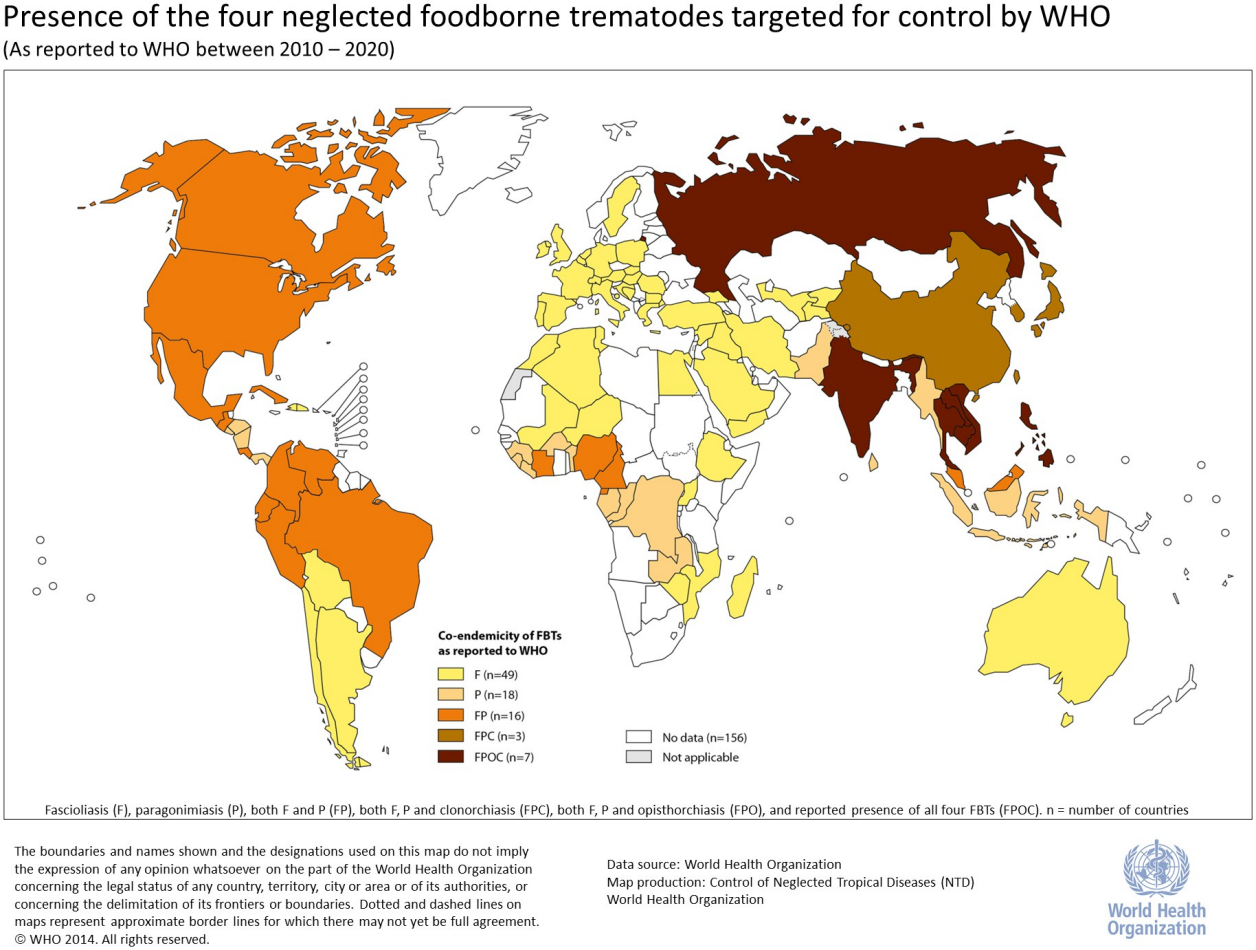
Map depicting the endemicity or co-endemicity of the 4 neglected FBTs as reported to the WHO between 2010 and 2019. The categories include fascioliasis (F); paragonimiasis (P); both F and P (FP); both F, P, and clonorchiasis (FPC); both F, P, and opisthorchiasis (FPO); and reported presence of all 4 FBTs (FPOC). The number of countries under each category is listed.

Countries reporting co-endemicity of FBTs to WHO included: (i) 16 countries reporting fascioliasis and paragonimiasis; (ii) 3 countries reporting fascioliasis, paragonimiasis, and clonorchiasis (China, Japan, and the Republic of Korea); and (iii) 7 countries reported all 4 FBTs (Cambodia, Lao People’s Democratic Republic, India, the Philippines, Russian Federation, Thailand, and Viet Nam) (Fig 2). Except for the United Republic of Tanzania and Kazakhstan, all countries that were included in the scoping review process also reported to WHO for at least 1 of the 4 FBTs during the 2010 to 2019 period. However, among these 26 countries with co-endemicity, only 3 countries (China, Republic of Korea, and Viet Nam) had conducted prevalence estimates for multiple FBTs in the published literature between 2010 and 2020.

### Fascioliasis

#### Environmental risk factors

Rural/agricultural environments were commonly identified risk factors for all geographical areas where fascioliasis was recorded. Rural and agricultural environments were often remote, with poor health care access, limited access to safe water, poor personal and environmental sanitation, and suitable intermediate and reservoir hosts to maintain transmission [17,18,24,26,27,31–33,35,39,43,47,46].

Suitable aquatic environments were identified as necessary for sustaining populations of snail intermediate hosts and maintaining the life cycle of *Fasciola* spp., making high rainfall areas, floodplains, and irrigated areas high-risk environments for *Fasciola* transmission [17,21,26,31,32,35,40,43,44,48,50,51].

High altitude, mountainous areas were identified as a risk factor for fascioliasis transmission in many records from the Region of the Americas, with hyperendemic areas identified in populations in higher altitude communities [24–29,31,32,132].

Areas with warm temperatures, irrigation, or environmental conditions resulting in high soil moisture favoured fascioliasis transmission in the Eastern Mediterranean Region, with the emergence of new disease foci observed in central and southwestern parts of the Islamic Republic of Iran [40,43,44,48,50,51].

#### Sociocultural risk factors

The consumption of raw vegetables and freshwater plants was a key risk factor in every geographical area where fascioliasis was reported.

Poor sanitation and hygiene was identified as a risk factor for all regions [18,19,21,24,26,28,29,31–33,35,37,42,47–51,54]. Poor water and sanitation included drinking of contaminated water, poor sanitation infrastructure, open defaecation, and poor food hygiene. Inadequate water, sanitation, and hygiene (WASH) contributed to ongoing transmission cycles, with poor sanitation resulting in contamination of water supplies that were used for drinking, cooking, and irrigation of crops [17,18,21,24,25,29,31,32,35,42,47,51,124].

Close contact with livestock was an important risk factor identified in the African, Americas, Eastern Mediterranean, and Western-Pacific regions, with free-roaming livestock and the use of animal manure on crops resulting in contamination of food and water sources [17,18,21,25,26,32,38,43,46,47,49–51,124].

Poverty and low socioeconomic settings were identified as risk factors in the Americas, Eastern Mediterranean, European, and Western Pacific regions [28,29,31,35,124].

#### Diagnostics

Faecal coprological tests were the commonly reported diagnostic method in all regions, except for the Eastern Mediterranean Region (represented primarily by the Islamic Republic of Iran), where serological methods were used for diagnosis in most of the included records [38–49].

Faecal parasitological tests were considered simple, cheap, and rapid for diagnosis and mass screening of populations. However, frequently identified challenges to faecal coprological tests included the low sensitivity of these tests due to low egg burdens, intermittent egg shedding, or acute infections, resulting in possible false negatives and underestimation of prevalence [19–21,26,28–30,32–34,47,77,81]. Increasing the number of faecal samples collected and increasing the number of slides examined from each sample were both identified as options to improve the sensitivity of these methods, but required further resources, had a lower compliance from patients, and faced logistical challenges with collection in remote areas [40,41,51].

The Eastern Mediterranean Region was the only region where serological diagnostic methods were more frequently used than faecal coprological tests and were considered more sensitive [40,48,49]. However, serological testing was unable to determine between past and current infections, and the possibility of cross-reactivity with other parasites could result in false positives [20,21,31,48,124]. These challenges meant that serological testing did not necessarily equate to true active cases and could overestimate *Fasciola* prevalence [31,45,124].

Using both parasitological and serological testing methods improved sensitivity and specificity of diagnosis; however, this was logistically challenging in many settings [21,25,35,41,124]. Coproantigen techniques were reported to be an easy and fast way in which to mass screen communities, allowing for rapid selective treatment, and reducing the probability of drug resistance [22].

#### Treatment and preventive measures

Treatment with triclabendazole was the most reported treatment for fascioliasis, although challenges to treatment included drug resistance, adverse drug reactions, and difficulty in obtaining the medication [18,20,29,33,34,47,81]. Four records reported the dosing regimen used for treatment, with 3 records using a single dose of 10 mg/kg [23,34,47] and the third record using 10 mg/kg per day for 2 days [81]. One record from Tanzania reported the use of nitazoxanide in areas with limited supply of triclabendazole, noting that not all patients were cleared with this treatment [18]. Another record reported the use of nitazoxanide in Mexico, noting its potential as an alternative to triclabendazole in countries where triclabendazole is not registered or where triclabendazole resistance is found [26].

Improved health education and awareness around disease transmission and risk factors was a commonly reported preventive measure, although it was noted that this needed to be implemented alongside improved WASH, disease surveillance, and improved access to diagnosis and treatment [17,33].

Management of reservoir species to interrupt transmission cycles was identified as an important preventive measure, with recommendations including surveillance and treatment of reservoir species, management of intermediate snail host populations, and grazing management of livestock, highlighting the need for an integrated approach to fascioliasis surveillance and management [20,21,25,27,31,38–40,43,45–49].

### Paragonimiasis

#### Environmental risk factors

The Three Gorges Reservoir area of China was reported to be area of high paragonimiasis endemicity, with hilly and forested areas, and natural bodies of water that sustain populations of both the intermediate snail host and intermediate freshwater crab host [93]. Mountainous areas of Vietnam were reported endemic and provided suitable habitats for intermediate mountainous crab hosts [128], while hyperendemic foci of paragonimiasis were identified in rural, remote hilly, and forested areas of northeastern India where populations had poor access to health care [53].

#### Sociocultural risk factors

The consumption of raw or undercooked crab was identified as a key risk factor in the Southeast Asia and Western Pacific regions [53,83,97,109–111,126], with the consumption of raw boar meat also identified as a risk factor in Japan [96]. Poverty and lack of education were identified as factors that contributed to high paragonimiasis prevalence in endemic areas of India [53].

#### Diagnostics

Reported diagnostic methods included examination of sputum and faeces for eggs, serological diagnosis, and intradermal testing. Serological methods were reported as a more sensitive diagnostic tool in both the African and the Southeast Asia regions, as ova were expectorated or shed intermittently in sputum and faeces, respectively [19,52]. Although positive serological results could include past infections and cross-reactions, it was considered a reliable diagnostic method [111]. Collection of multiple sputum samples increased diagnostic sensitivity of microscopy methods, with a record from the Philippines recommending 2 sputum samples to allow same day diagnosis, thereby improving patient compliance and reducing costs of diagnostics compared to samples collected over multiple days [97]. The same record recommended the collection of an early morning sputum sample followed by a spot sample, as the early morning sample improved sensitivity [97].

Co-endemicity and similarity of clinical symptoms with pulmonary tuberculosis, most notably a cough and hemoptysis, was a challenge for diagnosis in India and the Philippines [52,53,97]. Assumptions that these symptoms were indicative of tuberculosis contributed to underdiagnosis of paragonimiasis. These records recommended the integration of pulmonary tuberculosis and paragonimiasis surveillance and control programmes, noting that serological testing of symptomatic but tuberculosis negative patients, could improve paragonimiasis detection [52,53,97].

#### Treatment and preventive measures

Praziquantel was the only treatment reported, with 1 record reporting a treatment regime of 20 mg/kg per day for 6 days [83] and another reporting 75 mg/kg per day for 3 days [96].

Targeted active case detection and treatment of infected cases together with community education was found to reduce the prevalence of paragonimiasis in highly endemic areas of northeastern India [53].

Challenges to treatment included cases of underdosing reported in Japan, and a need for increased awareness among clinicians [96], and reinfection due to continued high-risk cultural food habits in Viet Nam despite more than 15 years of mass screening, treatment, and education [126].

### Clonorchiasis

#### Environmental risk factors

Rural/agricultural areas with poor sanitary conditions and river basin areas were identified as risk factors for clonorchiasis, as these environments supported both the intermediate freshwater snail host and the intermediate freshwater fish hosts [87,113,115,117].

#### Sociocultural risk factors

Consumption of raw freshwater fish was identified as the key risk factor for clonorchiasis, with reinfection in communities common due to deeply entrenched cultural food habits [87,117]. Poor sanitary conditions and food hygiene was also identified as a risk factor that could result in the contamination of food and utensils, leading to ingestion of metacercariae [87,88,90,117].

#### Diagnostics

Diagnosis by faecal parasitological methods were commonly reported, using either formalin-ether concentration technique (FECT) or the Kato–Katz thick smear method [122]. Such methods were widely used as they were simple, non-invasive, rapid, inexpensive, and were able to determine both the diagnosis and the intensity of infection [84,90,91]. However, microscopy methods could result in false negative results, particularly in light infections, and there were challenges in differentiating *C*. *sinensis* from other minute intestinal flukes [85,87,92,117]. Increasing the size of the faecal sample, repeating egg counts, and increasing the number of slides improved the sensitivity but also increased time and labour costs and was not always acceptable to communities [87].

Serological methods such as ELISA improved the sensitivity, but had poor specificity and were unable to detect early phases of infection [87]. Cross-reactivity with other parasites could also result in additional false-positive results [87,111]. However, while the ELISA method was recommended to be an auxiliary method to faecal microscopy for the diagnosis of individuals, ELISA methods were noted as being a potential option for large-scale screening to monitor community prevalence [87].

Molecular methods such as PCR were able to improve specificity and sensitivity but the high cost, need for trained personnel, and laboratory facilities, made this impractical for point-of-care diagnosis [87,90].

Several records noted that there may be value in using reports of raw freshwater fish consumption as a screening tool to identify high-risk communities, as this was simple, low cost, and had improved compliance from communities compared to faecal collection [92,117].

#### Treatment and preventive measures

Repeated mass drug administration (MDA) using praziquantel, combined with health education were commonly recommended prevention/treatment policies; however, reinfection was common due to the difficulty in changing deeply rooted cultural food habits [84,88,114,116]. Health education programmes that targeted community leaders were possibly more effective at promoting and maintaining changed behaviour in communities [116].

25 mg/kg every 5 h for 3 doses was the reported praziquantel dose regime by 4 records [92,116,117,129], with another record reporting a single treatment of 40 mg/kg [118]. Poor compliance with taking the second and third dose of praziquantel was identified as a possible factor for low cure rates in some communities [84]. Repeated mass or selective treatment every 6 to 12 months was recommended for reducing prevalence and reinfection in heavily endemic areas [8,115,119].

Reservoir hosts were an additional challenge to control, with diagnosed cases reported in dogs, cats, and pigs, and these reservoir hosts contributed to environmental contamination and sustained parasite lifecycle [84,89]. Treatment of reservoir hosts may contribute to reducing the source of infection in communities [84].

An integrated approach was recommended, with improved access to anthelmintics, improved health education, avoidance of raw fish consumption, active screening for early disease detection, treatment of reservoir hosts, and elimination of intermediate host snails [84,114,115].

### Opisthorchiasis

#### Environmental risk factors

River basins were reported risk areas for the transmission of *Opisthorchis* species, as these environments supported both the snail and fish intermediate hosts [37,60]. Similarly, areas of higher rainfall and rural, lowland villages with surrounding land that was dominated by high water content (wetlands, paddies, streams, ponds, and lakes) were reported to have high prevalence of *O*. *viverrini* due to the suitable environments for intermediate hosts [60,99,103]. The development of water resources for aquaculture and irrigation may have contributed to the high prevalence found in northeast Thailand [60]. Higher altitude areas were reported to have a lower risk of opisthorchiasis, although this was likely related to accessibility to freshwater fish and the difference of cultural food preferences [99].

High infection rates were reported for *O*. *felineus* in Western Siberia [37] and northern Kazakhstan [36], while high prevalence of *O*. *viverrini* was reported throughout the Mekong Basin in Southeast Asia, including endemic areas in north and northeast Thailand, central and southern Lao PDR, southern Cambodia, and southern Viet Nam [54,56,57,59,60,62,63,79].

#### Sociocultural risk factors

The consumption of raw or undercooked freshwater fish was the most commonly reported risk factor for infection with *Opisthorchis* spp., and this food preference had a strong cultural basis contributing to frequent reinfection [37,54,58,60,64,65,67,80,99].

Cultural habits that may contribute to transmission include the eating of food with fingers, potentially resulting in hand contamination [58], and traditional food sharing habits that was reported to result in clusters of opisthorchiasis infection in villages [65].

Poor food hygiene and sanitation was reported to result in contamination of other food, surfaces, and utensils [54,106]. Several records reported poor access to toilets and defaecation in fields, resulting in contamination of water bodies and facilitating ongoing transmission in communities [59,60,80,99,100].

Free-roaming domestic cats and dogs were reported to act as reservoir hosts, with dogs often accompanying owners to rice fields, contributing to maintenance of transmission cycles in communities [105].

Lower levels of education was associated with higher risk of infection [64,66].

#### Diagnostics

Similar to the other FBTs reported, diagnosis of opisthorchiasis was primarily based on the detection of eggs from faecal samples with microscopy, as this was a low cost and non-invasive method [54,56,59,67,99]. However, this had limited diagnostic sensitivity and specificity, required skill parasitologists for diagnosis, and it was difficult to differentiate opisthorchiid eggs from other small intestinal flukes [54,63,67,80,99,103,105,106]. Additional smears from multiple stool samples were reported to improve sensitivity [99,100,103,105,106].

Diagnosis using a monoclonal antibody-based enzyme-linked immunosorbent assay for measuring OV excretory-secretory antigens in urine was discussed as a potential diagnostic method, as this was non-invasive and could be useful for mobile screening [67].

One record reported the use of an *O*. *viverrini* verbal screening test, which included questions around the consumption of raw fish [56]. This method was a simple, low-cost, and rapid screening test that may be of use for identifying at risk populations and screening for large-scale prevention and control [56].

#### Treatment and preventive measures

Treatment consisted of praziquantel, using a single dose of 40 mg/kg [62,64,65,67,75,80,99,102,117,127], with 1 record reporting 25 mg/kg for 3 doses over a single day [70]. Limited awareness about the severity of opisthorchiasis, community complacency driven by the belief that symptoms were mild and an effective treatment was available, and deeply rooted cultural food habits contributed to frequent reinfection in endemic communities [59,62,67,71,73,74,100,106]. It was also noted that repeated cycles of reinfection and treatment with praziquantel could result in drug resistance and potentially increase the risk of cholangiocarcinoma [77].

Mass drug administration combined with culturally appropriate health education around food consumption attitudes, personal hygiene, and sanitation was noted to be important in reducing transmission and reinfection [58,59,62,99].

Several records noted that there were high levels of infection in reservoir species, including free-roaming cats and dogs, which were contributing to community transmission [64]. Recommendations for opisthorchiasis control included the treatment of reservoir species in an integrated approach to break the epidemiological cycle and improve the effectiveness of interventions [62,64,102,105].

Access to sanitation was reported to be protective, as this reduced environmental contamination and infection intensity; however, further awareness building around sanitation was required as often latrines were not used even if these were available [100].

## Discussion

There was a mismatch between the data that are reported to WHO and that which are reported in the literature reviewed, with the data captured in this review representing 25 countries despite 93 countries reporting FBT “presence only” data to WHO between 2010 and 2019. Similarly, although 26 countries reported ≥ 2 FBTs to WHO, only 3 countries in this review conducted studies for multiple FBTs (*Fasciola*, *Paragonimus*, *Clonorchis* in China; *Paragonimus* and *Clonorchis* in Republic of Korea; all FBTs in Viet Nam). This mismatch of data highlights the limited availability of reliable data in some regions and challenges in reporting FBTs and the need for more research in many geographical areas. Additional countries were identified during the screening process of records that reported clinical cases of FBTs, without reporting prevalence, further enforcing the need to instal surveillance and mapping where cases are reported.

Although the geographical distribution of each FBT discussed in this review reflects what has previously been reported, there were differences in reported prevalences. The highest FBT study prevalence was reported for *O*. *viverrini* in Lao People’s Democratic Republic. This is consistent with the reported high prevalence of 37.02% by Furst and colleagues; however, the records in our review identified study prevalences as high as 88.7% in some areas [6,100]. While this could be due to increasing cases or improved surveillance resulting in increased case detection, the prevalences depicted in this review are based on very limited data and are unlikely to reflect true national prevalence. The highest fascioliasis study prevalence identified in this review was in the Region of the Americas, consistent with previous literature [132]. A high study prevalence was also identified in the United Republic of Tanzania, despite no reporting of FBTs to WHO in this time period [18]. The highest recorded study prevalence of paragonimiasis was also reported in the African Region, in Cameroon [18]. The available data on FBTs in Africa is limited, and the reports of high prevalences for both fascioliasis and paragonimiasis support an urgent need for research to better understand the epidemiology and burden of FBTs in this region.

Several limitations exist with our method of reporting prevalence. Although no language restrictions were set, all search terms were in English, potentially resulting in the exclusion of records from areas that have a high burden of FBTs, including Latin America and the Russian Federation. This bias is most notable with the absence of records for *F*. *Hepatica* in the Americas, despite the well-documented hyperendemic areas in this region [132]. Due to the limited availability of national prevalence data, we instead extracted the study prevalence from each record, using the mean study prevalence when multiple prevalences were recorded. However, as study size and methodology varied greatly between records, and there was a wide range of study prevalences reported even between geographically close areas with similar risk factors, this data cannot be used to reliably estimate national prevalence.

Nevertheless, the data gaps recognised here demonstrate the need for continued improvement of mapping and surveillance to confirm focal points of disease that should be targeted for public health interventions. While disease burden can be estimated within assumptions and available data [6], the limited knowledge available on FBTs in many areas highlights the need for more research and the promotion of successful frameworks, guidelines, and control programmes for surveillance and reporting of FBTs. For example, initiatives in highly endemic fascioliasis areas of Bolivia [133–139], Peru [22,140], Argentina [141], Egypt [142], Pakistan [143] and Vietnam [144], and highly endemic opisthorchiasis areas of Thailand [145–147] have included experimental studies and field surveys to assess transmission and infection sources, field evaluation of diagnostic tools, passive and active surveillance, mass chemotherapy, and the promotion of health awareness among a wide variety of stakeholders. Comprehensive education programmes aimed at community leaders and schoolchildren have been implemented in China [148] and the Republic of Korea [116] to complement mass screening and MDA for clonorchiasis, and reduce transmission and infection risk, and studies in the Philippines have explored the integration of paragonimiasis surveillance and control with tuberculosis control to improve finding and treatment of cases [97,149,150]. These efforts have helped to improve surveillance and case detection [22,135], map FBT endemic areas [140], understand and reduce transmission and infection risk [138,139,147], lower FBT prevalence in humans and intermediate hosts [116,147], and helped to prioritise where resources should be most effectively used in control programmes [135,136,139]. Such initiatives could help inform future studies in other endemic areas to identify within country variation of the endemic areas and delineate treatment strategies according to the level of geographical co-endemicity.

Several themes were highlighted from the qualitative data extracted from the literature; however; as these data reflect what each record discussed and were not necessarily supported by strong epidemiological data, the trends identified in this review may not align with the true epidemiological situation in communities. In addition to this, as our search strategy focused on human prevalence studies, prevalence studies of other species were not captured, and the extracted qualitative data does not take into account the evidence provided in many valuable epidemiological or transmission risk studies available. This is a significant limitation in our review of qualitative data, and a more comprehensive review focusing on such qualitative data, and prevalence in intermediate and reservoir hosts should be considered to capture these aspects. The inclusion of robust epidemiological data in future prevalence studies would also be valuable to support ongoing surveillance and targeted prevention and treatment programmes.

Despite the epidemiological differences between FBTs, there was significant overlap in the geographical and sociocultural factors that promoted infection and sustained transmission for each FBT. Rural and agricultural communities with appropriate aquatic environments provided a suitable ecological niche for intermediate snail hosts, and secondary fish and crustacean hosts, and also promoted activities that increased the risk of exposure to FBTs (poor sanitation, high-risk food consumption, contact with livestock). Identifying communities that meet these risk criteria can help predict distribution at non-surveyed locations, inform disease surveillance, and help target control programmes against multiple FBTs [99]. However, the interplay of these factors is complex, and endemic and non-endemic villages often coexist within close proximity despite meeting the same risk criteria [80]. The wide range of study prevalences identified in this review, even between geographically close areas with similar risk factors, highlights the need for local health systems to be strengthened and engaged in order for FBTs to be prioritised and dealt with by local health authorities [97,104]. Improved screening is needed to confirm these focal areas of endemicity and co-endemicity. As recognised by previous studies, this review also highlighted the logistical and diagnostic challenges of available methods and the need for an efficient and accurate diagnostic methods to improve surveillance [18]. Although MDA and improved community awareness were frequently discussed as preventive factors for FBT infection, this review highlighted that even in communities that received regular treatment with anthelmintics and were aware of the risks of infection, there continued to be ongoing community transmission and reinfection [80,128,131]. Communities with strong cultural dietary habits and poor sanitation will continue to have a high risk of exposure to infection [17,58]. High infection rates in untreated domestic reservoir species will contribute to ongoing transmission, as well as resulting in veterinary and economic impacts in communities that are dependent on agriculture for their livelihoods [2,17,25,64,105].

One Health approaches are needed to reduce environmental contamination, improve access to clean water and adequate sanitation, and address the role of reservoir hosts in FBT transmission [81,104,105]. One Health is defined as an “integrated, unifying approach that aims to sustainably balance and optimise the health of people, animals, and ecosystems,” and is core to the NTD road map [151,152]. However, while the concept of One Health is becoming more familiar, there is still significant work to be done to promote a consistent understanding across sectors and finding practical ways to operationalise One Health in disease prevention and control programmes. One Health approaches need to address more than the zoonotic pathway of disease transmission, but should also promote coordinated resource allocation and planning, and explore opportunities for treatment implementation to be integrated with that of other diseases and protocols. Implementing such approaches, at both local and national levels, are integral to effectively and sustainably reducing the burden of FBTs. These approaches should further be integrated into Universal Health Care programmes to ensure equitable implementation of the road map for NTD control by 2030 [153]. Examples of successfully implemented One Health approaches have been demonstrated in several areas, including *Fascioliasis* endemic areas of Northern Bolivian Altiplano and *Opisthorchiasis* endemic areas of Khon Kaen Province in Thailand, and such examples can provide a framework that can help build similar One Health programmes in other endemic areas.

## Conclusions

FBTs are targeted for control as part of the WHO NTD road map 2021–2030, with mapping and surveillance and capacity, awareness, and advocacy building identified as critical actions required to reach the 2030 targets. FBTs, like other NTDs, impact the most impoverished populations and lack the surveillance systems and tools to adequately ascertain their true burden. The continued high burden of FBTs identified in this review, and the mismatch between the data that are reported to WHO and that which are reported in the literature, demonstrates the need for programmes to improve mapping and surveillance in order to target public health interventions. Integrated One Health approaches across environmental, animal, and human health sectors are needed to meet the 2030 goals.

## Supporting information

S1 Supplementary material Table A in S1 Supplementary material. Box A in S1 Supplementary material: Search terms.
(DOCX)
