# Supplementary material for: Global prevalence of 4 neglected foodborne trematodes targeted for control by WHO: A scoping review to highlight the gaps
Source: PLoS Negl Trop Dis. 2023 Mar 2;17(3):e0011073. doi: 10.1371/journal.pntd.0011073 (PMC9980766; doi:10.1371/journal.pntd.0011073)
Supplement: S1 Supplementary material — Table A in S1 Supplementary material. Box A in S1 Supplementary material: Search terms. (DOCX) [file pntd.0011073.s001.docx]

Table A in S1 Supplementary Material

| Country/ territory | Sovereign | WHO Region | Reported to WHO during 2010 - 2019 | Reported in the literature 2019-2020 |
| --- | --- | --- | --- | --- |
| Algeria | Algeria | AFR | F |  |
| Argentina | Argentina | AMR | F | F |
| Australia | Australia | WPR | F |  |
| Austria | Austria | EUR | F |  |
| Belgium | Belgium | EUR | F |  |
| Bolivia (Plurinational State of) | Bolivia (Plurinational State of) | AMR | F | F |
| Bosnia and Herzegovina | Bosnia and Herzegovina | EUR | F |  |
| Bulgaria | Bulgaria | EUR | F |  |
| Chile | Chile | AMR | F |  |
| Croatia | Croatia | EUR | F |  |
| Dominican Republic | Dominican Republic | AMR | F |  |
| Egypt | Egypt | EMR | F |  |
| Ethiopia | Ethiopia | AFR | F | F |
| France | France | EUR | F |  |
| Germany | Germany | EUR | F |  |
| Greece | Greece | EUR | F |  |
| Haiti | Haiti | AMR | F | F |
| Hungary | Hungary | EUR | F |  |
| Iran (Islamic Republic of) | Iran (Islamic Republic of) | EMR | F | F |
| Iraq | Iraq | EMR | F |  |
| Ireland | Ireland | EUR | F |  |
| Italy | Italy | EUR | F |  |
| Kyrgyzstan | Kyrgyzstan | EUR | F | F |
| Madagascar | Madagascar | AFR | F |  |
| Mali | Mali | AFR | F |  |
| Malta | Malta | EUR | F |  |
| Morocco | Morocco | EMR | F |  |
| Mozambique | Mozambique | AFR | F |  |
| Netherlands | Netherlands | EUR | F |  |
| Niger | Niger | AFR | F |  |
| Poland | Poland | EUR | F |  |
| Portugal | Portugal | EUR | F |  |
| Romania | Romania | EUR | F |  |
| San Marino | San Marino | EUR | F |  |
| Saudi Arabia | Saudi Arabia | EMR | F |  |
| Slovakia | Slovakia | EUR | F |  |
| Spain | Spain | EUR | F |  |
| Sweden | Sweden | EUR | F |  |
| Switzerland | Switzerland | EUR | F |  |
| Syrian Arab Republic | Syrian Arab Republic | EMR | F |  |
| Tajikistan | Tajikistan | EUR | F |  |
| Tunisia | Tunisia | EMR | F |  |
| Turkey | Turkey | EUR | F | F |
| Uganda | Uganda | AFR | F |  |
| United Kingdom of Great Britain and Northern Ireland | United Kingdom of Great Britain and Northern Ireland | EUR | F |  |
| Uzbekistan | Uzbekistan | EUR | F |  |
| Yemen | Yemen | EMR | F |  |
| Zimbabwe | Zimbabwe | AFR | F |  |
| Brazil | Brazil | AMR | FP | F |
| Côte d'Ivoire | Côte d'Ivoire | AFR | FP |  |
| Cameroon | Cameroon | AFR | FP | P |
| Canada | Canada | AMR | FP |  |
| Colombia | Colombia | AMR | FP |  |
| Costa Rica | Costa Rica | AMR | FP |  |
| Cuba | Cuba | AMR | FP | F |
| Ecuador | Ecuador | AMR | FP |  |
| Equatorial Guinea | Equatorial Guinea | AFR | FP |  |
| Guatemala | Guatemala | AMR | FP |  |
| Malaysia | Malaysia | WPR | FP |  |
| Mexico | Mexico | AMR | FP | F |
| Nigeria | Nigeria | AFR | FP |  |
| Peru | Peru | AMR | FP | F |
| United States of America | United States of America | AMR | FP |  |
| Venezuela (Bolivarian Republic of) | Venezuela (Bolivarian Republic of) | AMR | FP |  |
| China | China | WPR | FPC | FPC |
| Japan | Japan | WPR | FPC | P |
| Republic of Korea | Republic of Korea | WPR | FPC | PC |
| Cambodia | Cambodia | WPR | FPOC | O |
| Lao People's Democratic Republic | Lao People's Democratic Republic | WPR | FPOC | O |
| India | India | SEAR | FPOC | P |
| Philippines | Philippines | WPR | FPOC | P |
| Russian Federation | Russian Federation | EUR | FPOC | O |
| Thailand | Thailand | SEAR | FPOC | O |
| Viet Nam | Viet Nam | WPR | FPOC | FPOC |
| Benin | Benin | AFR | P |  |
| Burkina Faso | Burkina Faso | AFR | P |  |
| Congo | Congo | AFR | P |  |
| Democratic Republic of the Congo | Democratic Republic of the Congo | AFR | P |  |
| El Salvador | El Salvador | AMR | P |  |
| Gabon | Gabon | AFR | P |  |
| Georgia | Georgia | EURO | F |  |
| Guinea | Guinea | AFR | P |  |
| Honduras | Honduras | AMR | P |  |
| Indonesia | Indonesia | SEAR | P |  |
| Liberia | Liberia | AFR | P |  |
| Myanmar | Myanmar | SEAR | P | O |
| Nicaragua | Nicaragua | AMR | P |  |
| Pakistan | Pakistan | EMR | P | F |
| Panama | Panama | AMR | P |  |
| Papua New Guinea | Papua New Guinea | WPR | P |  |
| Sierra Leone | Sierra Leone | AFR | P |  |
| Sri Lanka | Sri Lanka | SEAR | P |  |
| Zambia | Zambia | AFR | P |  |

**United Republic of Tanzania did not report any presence only data during the captured time period to WHO, although Fasciola was recorded in the literature. Kazakhstan did not report any presence only data during the captured time period to WHO, although Opisthorchis was recorded in the literature.*

*Countries that did not report to WHO and were not identified to conduct research on the 4 FBTs were removed from the list here for brevity*

Box A in S1 Supplementary Material

*Search terms*

- Fasciola AND (treat* OR detect*)
- Fasciola AND burden
- Fasciola AND (prevalence OR case* OR incidence)
- Clonorchis AND (treat* OR detect*)
- Clonorchis AND burden
- Clonorchis AND (prevalence OR case* OR incidence)
- Paragonimus AND (treat* OR detect*)
- Paragonimus AND burden
- Paragonimus AND (prevalence OR case* OR incidence)
- Opisthorchis AND (treat* OR detect*)
- Opisthorchis AND burden
- Opisthorchis AND (prevalence OR case* OR incidence)
- Foodborne trematod* AND (treat* OR detect*)
- Foodborne trematod* AND burden
- Foodborne trematod* AND (prevalence OR case* OR incidence)

Box B in S1 Supplementary Material

*Exclusion criteria*

- No human population identified
- Record did not include any of the four FBTs discussed
- No geographical area was identified
- No diagnostic method was identified
- No prevalence/incidence was reported
- Literature reviews/commentaries (*one review of primary medical data for the Russian Federation was included)
